# Characterization of the complete mitochondrial genome of *Pentatoma semiannulata* (Hemiptera: Pentatomidae)

**DOI:** 10.1080/23802359.2021.1875912

**Published:** 2021-03-11

**Authors:** Juan Wang, Yutong Ji, Hu Li, Fan Song, Lisheng Zhang, Mengqing Wang

**Affiliations:** a Institute of Plant Protection, Chinese Academy of Agricultural Sciences, Beijing, China; b Department of Entomology, MOA Key Lab of Pest Monitoring and Green Management, College of Plant Protection, China Agricultural University, Beijing, China

**Keywords:** Mitochondrial genome, Heteroptera, Pentatominae, *Pentatoma semiannulata*

## Abstract

The *Pentatoma semiannulata* is an important fruit pest in Chinese agricultural system. In current study, the complete mitochondrial genome of *P. semiannulata* is determined. This mitogenome is 15,515 bp in size and comprises of 13 protein-coding genes, 22 transfer RNA genes, two ribosomal RNA genes, and a control region. Gene order is identical to that of the putative ancestral arrangement of insects. All protein-coding genes initiate with ATN, except for *ATP8*, *COX1* and *NAD1* use GTG or TTG as the start codon, and terminate with TAA with the exception for *COX2* which uses a single T residue as the stop codon. All tRNAs, ranging from 62 to 72 bp, have the clover-leaf structure except for *tRNA^Ser(AGN)^*. The monophyly of Pentatomidae is highly supported by the phylogenetic tree and *P. semiannulata* is very close to other herbivorous species of the remaining Pentatomidae species.

Pentatomidae is one of the most diverse groups in Heteroptera, occurring world widely (Rider et al. 2018). *Pentatoma semiannulata* belongs to the subfamily Pentatominae in Pentatomidae, has gotten farmers’ much attention by its harm to pear and birch. In this study, the complete mitochondrial genome of *P. semiannulata* was sequenced and described. Adult specimens were collected from Ningshan County (28°26′2″N, 108°26′52″E) of Shaanxi Province in China in 2018. Samples have been deposited at the Entomological Museum of China Agricultural University (No. HEM-1732).

The total genomic DNA was extracted from the whole body of the specimen using the QIAamp DNA Blood Mini Kit (Qiagen, Germany) and stored at −20 °C until needed. The mitogenome was sequenced in BeiRuiHeKang biotechnology company used NGS. An Illumina TruSeq library was prepared and sequenced using the Illumina Novaseq PE150 platform with 150 bp paired-end reads. Raw reads, low quality and short reads were trimmed and removed (Schmieder and Edwards 2011; Bolger et al. 2014). High quality reads were then used to produce a denovo assembly using IDBA-UD (Peng et al. 2012) with minimum and maximum k values of 40 and 160 bp, respectively. The accuracy of the assembly was verified by mapping clean reads onto the obtained mt contig (mismatches = 2%, maximum gap size = 3 bp and minimum overlap = 100 bp).

The complete mitogenome of *P. semiannulata* is 15,515 bp in size (GenBank accession number: MT985377) including 37 typical insect mitochondrial genes (13 protein-coding genes, 22 transfer RNA genes, and two ribosomal RNA genes) and a control region. Gene order is identical to that of the putative ancestral arrangement of insects (Cameron 2014; Xu et al. 2019). The nucleotide composition of the mitogenome is biased toward A and T, with 77.1% of A + T content (A = 42.2%, T = 34.9%, C = 12.8%, G = 10.1%). The AT-skew is positive (0.09) whereas GC-skew is negative (−0.12). Ten protein-coding genes initiate with ATN codons (three with ATA, four with ATG, two with ATT, and one with ATC), whereas *ATP8* starts with GTG and TTG is used by *COX1* and *NAD1* as the start codon. The stop codon TAA or TAG is distributed to twelve protein-coding genes (eleven with TAA, and one with TAG). However, the *COX2* uses a single T residue as an incomplete stop codon which is common in other true bug mitogenomes (Wang et al. 2017; Zhao et al. 2017; Zhang et al. 2019; Wu et al. 2020a).

There are 22 tRNA genes, ranging from 62 to 72 bp in length, and all of them can be folded into typical clover-leaf secondary structure except for *tRNA^Ser(AGN)^*, the dihydrouridine (DHU) arm of which forms a loop, as is common phenomenon in most insects (Jiang et al. 2016; Xu et al. 2019; Wu et al. 2020a, 2020b). The length of *IrRNA* and *srRNA* is 1,273 bp and 829 bp, respectively. The A + T content of *IrRNA* and *srRNA* are 79.7% and 76.7%. The control region is located between *srRNA* and *tRNA^IIe^*, which is 782 bp in length with an A + T content of 74.2%.

Phylogenetic tree was constructed by maximum-likelihood (ML) analysis and generated by IQ-TREE 2.0.6 (Bui et al. 2020), based on the dataset of the 13 protein-coding genes and two rRNA genes from 12 species of different families and two outgroups (Figure 1). Each family showed a monophyletic cluster. The monophyly of the Pentatomidae was highly supported in this phylogenetic analysis, and predatory species were evolved from herbivorous species in the family Pentatomidae. The complete mitogenome of *P. semiannulata* could provide the molecular genetic markers for the further phylogenetic analysis in Pentatomidae.

**Figure 1.**
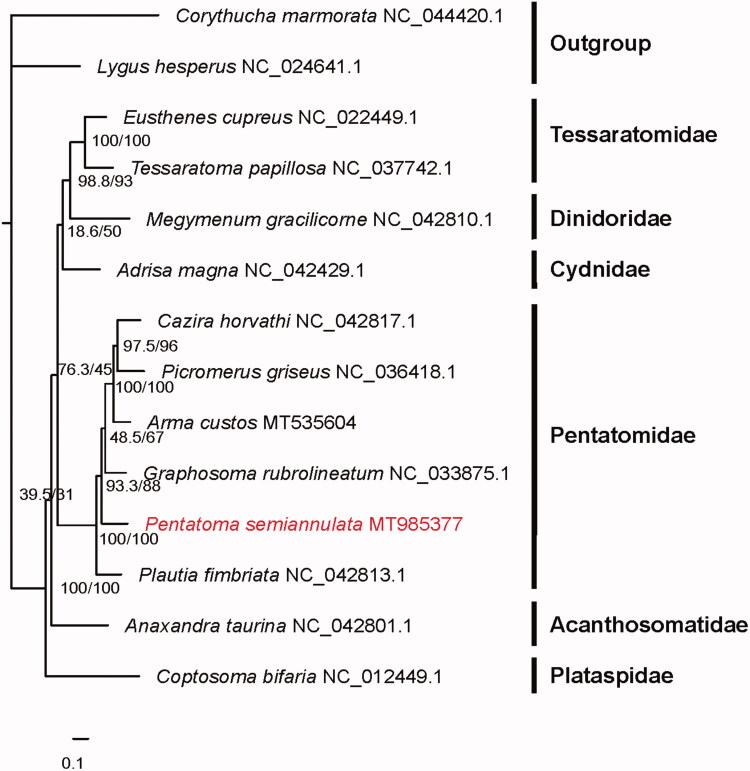
Phylogeny of *Pentatoma semiannulata* and other 11 Pentatomoidea species which was inferred from ML analysis of the 13 protein-coding genes and two rRNAs genes. Numbers above each node separated by ‘/’ indicated support values of SH-aLRT (left) and ultrafast bootstrap (right) respectively. The newly sequenced mitochondrial genome was highlighted in red.

## Data Availability

The data that support the findings of this study are openly available in [NCBI] at [https://www.ncbi.nlm.nih.gov/], reference number [MT985377].
